# Zonisamide Ameliorates Microglial Mitochondriopathy in Parkinson’s Disease Models

**DOI:** 10.3390/brainsci12020268

**Published:** 2022-02-14

**Authors:** Satoshi Tada, Mohammed E. Choudhury, Madoka Kubo, Rina Ando, Junya Tanaka, Masahiro Nagai

**Affiliations:** 1 Department of Clinical Pharmacology and Therapeutics, Ehime University Graduate School of Medicine, Toon 791-0295, Ehime, Japan; tada.satoshi.ia@ehime-u.ac.jp (S.T.); mkubo@m.ehime-u.ac.jp (M.K.); ando.rina.cn@ehime-u.ac.jp (R.A.); 2 Department of Molecular and Cellular Physiology, Graduate School of Medicine, Ehime University, Toon 791-0295, Ehime, Japan; mechoudh@m.ehime-u.ac.jp (M.E.C.); jtanaka@m.ehime-u.ac.jp (J.T.)

**Keywords:** zonisamide, microglia, inflammation, mitochondria, Parkinson’s disease

## Abstract

Mitochondrial dysfunction and exacerbated neuroinflammation are critical factors in the pathogenesis of both familial and non-familial forms of Parkinson’s disease (PD). This study aims to understand the possible ameliorative effects of zonisamide on microglial mitochondrial dysfunction in PD. We prepared 1-methyl-4-phenyl-1,2,3,6-tetrahydropyridine and lipopolysaccharide (LPS) co-treated mouse models of PD to investigate the effects of zonisamide on mitochondrial reactive oxygen species generation in microglial cells. Consequently, we utilised a mouse BV2 cell line that is commonly used for microglial studies to determine whether zonisamide could ameliorate LPS-treated mitochondrial dysfunction in microglia. Flow cytometry assay indicated that zonisamide abolished microglial reactive oxygen species (ROS) generation in PD models. Extracellular flux assays showed that LPS exposure to BV2 cells at 1 μg/mL drastically reduced the mitochondrial oxygen consumption rate (OCR) and extracellular acidification rate (ECAR). Zonisamide overcame the inhibitory effects of LPS on mitochondrial OCR. Our present data provide novel evidence on the ameliorative effect of zonisamide against microglial mitochondrial dysfunction and support its clinical use as an antiparkinsonian drug.

## 1. Introduction

Parkinson’s disease (PD) is the second most common neurodegenerative disease, characterised by a progressive loss of dopaminergic neurones in the substantia nigra pars compacta [1]. Microglial cells are central to the pathophysiology of PD because they are potentially harmful to neurones when activated. Overwhelmed microglia can produce a range of reactive oxygen species (ROS), including nitric oxide and superoxide anions, and release proinflammatory cytokines that exacerbate dopaminergic degeneration and neurologic deficits in neurodegenerative diseases [2]. With respect to microglial phagocytosis, 1-methyl-4-phenyl-1,2,3,6-tetrahydropyridine (MPTP)-treated mice show a milieu of microglial phagocytosis, where microglia polarise and approach damaged dopaminergic neurones for phagocytosis [3]. Increased microglial phagocytosis is related to PD progression [4], and blocking microglial phagocytosis can rescue live neurones from inflammation-mediated neuronal death [5]. The expression of CD68, a microglial phagosome marker, has been found in the substantia nigra of patients with PD and PD animal models [6,7]. A recent study demonstrated that microglia depletion with CSF-1R inhibitors (GW2580) attenuated MPTP-induced dopaminergic neuronal loss and motor behavioural deficits [8]. Another study showed that a CSF-1R inhibitor (PLX3397) caused marked microglial ablation and ameliorated motor deficits in a transgenic mouse model of PD [9]. In contrast, PLX3397 exacerbates impaired motor activity, loss of dopaminergic neurones, and locomotor behavioural abnormalities [10]. In regard to microglia and inflammation, many cellular and animal studies using anti-inflammatory drugs have shown potentially ameliorative effects on PD symptoms, but their clinical use in terms of decelerating the progression of PD remains elusive [11]. Therefore, it is essential to identify a potent agent for microglial phenotype remodelling that is of important clinical use as an antiparkinsonian drug.

Zonisamide (ZNS) has beneficial effects on motor symptoms and sleep disorders in levodopa-treated patients with PD [12,13]. In our previous studies, ZNS showed an ameliorative effect against MPTP and a 6-OHDA-induced PD models of common marmosets, mice, and rats [14,15,16,17]. A recent post-mortem study on patients with PD showed that ZNS could suppress microglial Nav 1.6 [18], which has been demonstrated to be a significant contributor to microglial activation [19,20,21]. Additionally, ZNS improved neuropathic pain by inhibiting microglial activation in the spinal cord of a mouse model [22]. However, current studies have not revealed any evidence regarding the effects of ZNS on switching microglial functions in the milieu of PD. Therefore, we assess the effects of ZNS on mitochondrial activity using inflammatory in vivo and in vitro PD models. Considering the potential role of impaired microglial phagocytosis in the pathogenesis of PD [23], we specifically focus on the link between mitochondrial dysfunction and impaired phagocytosis in PD scenarios.

## 2. Materials and Methods

### 2.1. Animals

All experiments were performed in accordance with the guidelines of the Ethics Committee for Animal Experimentation of Ehime University, Japan. C57/BL6 mice (Clea Japan, Tokyo) were purchased, bred, and housed (4 mice/cage) at a temperature of 25 ± 1 °C, with a relative humidity of 55% ± 5%, under a 12 h light (7:00–12:00)/12 h dark (19:00–7:00) cycle of automatic illumination at the Animal facility, Advanced Research Support Centre, Ehime University. For the present study, we selected mice that were 9 ± 0.5 weeks old with a body weight of 25 ± 2 g.

### 2.2. Cells

The murine microglial cell line BV2 and the neuronal cell line Neuro 2A were purchased from ATCC (Manassas, VA, USA). Both cell lines were maintained in DMEM (Wako, Osaka, Japan) supplemented with 10% foetal calf serum (Biowest, Nuaille, France) and antibiotics (Wako).

### 2.3. Flow Cytometry

**Figure 1:**
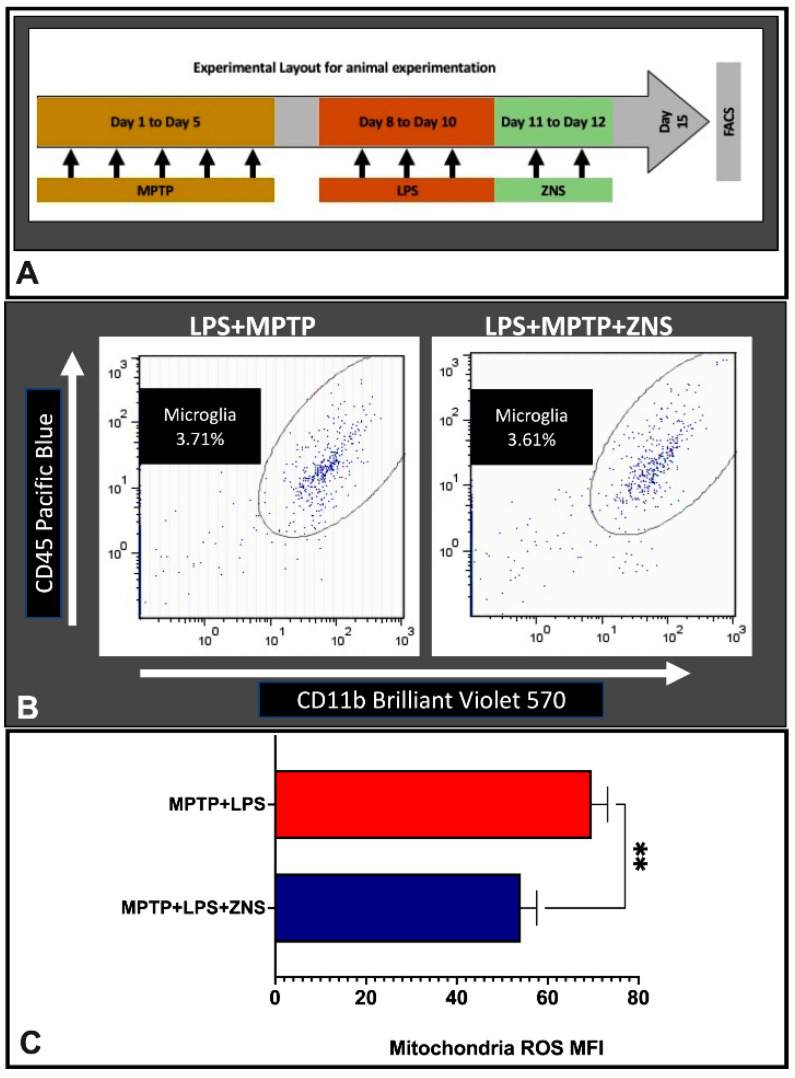
Effects of ZNS in MPTP and LPS-induced mice model of Parkinsonism. (**A**) Timeline of experiments. (**B**) Gating strategy and representative dot plot data where microglial cells were gated with CD45 and CD11b double-positive cells. (**C**) mitROS generation was assessed on microglia cell and presented with MFI where ZNS (40 mg/kg) suppressed microglia-derived mitROS in MPTP (25 mg/kg) and LPS (100 µg/kg) treated Parkinson’s disease model mice. Data from four mice for each group were expressed as mean ± SD. Asterisks indicate ** 0.01, by student *t*-test. MPTP, 1-methyl-4-phenyl-1,2,3,6-tetrahydropyridine; LPS, lipopolysaccharide; FACS, fluorescence activated cell sorting; ZNS, zonisamide; ROS, reactive oxygen species; mitROS, mitochondrial reactive oxygen species; MFI, mean fluorescence intensity; SD, standard deviation.

For the in vivo study, mice received subcutaneous injections of MPTP (Sigma-Aldrich, St. Louis, MO, USA) and lipopolysaccharide (LPS) (Sigma-Aldrich), as described in Figure 1A. Mice were sacrificed under deep anaesthesia (CO_2_ exposure, Matsuyama Nishi Sanso Company, Matsuyama, Japan), and whole brains were dissected out and subjected to flow cytometry analysis for multicolour immunofluorescence immuno-labelling (Brilliant Violet 570™ anti-mouse/human CD11b Antibody and Pacific Blue™ anti-mouse CD45 Antibody, BioLegend, San Diego, CA, USA) and mitochondrial ROS (MitoROS 520 (AAT Bioquest, Sunnyvale, CA, USA)) as described in earlier studies [24,25]. For the in vitro study, BV2 cells were exposed with or without LPS (1 μg/mL) and incubated with or without ZNS (100 μM) for 24 h. The cells were then processed for phagocytosis and mitROS assays as described in earlier studies [24,25]. The Gallios instrument (Beckman-Coulter, Brea, CA, USA) was used to perform flow cytometry of cells, and the results were analysed using FlowJo (Becton, Dickinson and Company, Franklin Lakes, NJ, USA).

### 2.4. Mitochondrial Bioenergetic Assay

The oxygen consumption rate (OCR) and extracellular acidification rate (ECAR) were measured to assess mitochondrial function in BV2 and Neuro 2A cells using the Seahorse XFp Extracellular Flux Analyser and XFp mito stress test kit (Agilent Technologies, Santa Clara, CA, USA), as described in an earlier study [24].

### 2.5. Quantitative Real-Time RT-PCR (qPCR)

Total RNA was extracted from cells using the Maxwell^®®^ 16 Cell LEV Total RNA Purification Kit (Promega, Madison, WI, USA), and cDNA was synthesised using ReverTra Ace™ qPCR RT Master Mix with gDNA Remover (Toyobo, Osaka Japan). The cDNA samples were prepared from four separate culture samples and qPCR was performed as described before [26]. The primer sequences used in this study were purchased from (Hokkaido System Science Co., LTD, Hokkaido, Japan) and are listed as follows: Timm23, forward (TATGGTGACTAGGCAAGGAG) and reverse (GCTACTGTGTTGAGGTCATC); HIF-1α, forward (TAAATGTTCTGCCCACCCT) and reverse (GCGACAAAGTGCATAAAACC); and GAPDH, forward (ACCCAGAAGACTGTGGATGG) and reverse (CACATTGGGGGTAGGAACAC).

### 2.6. Statistical Analysis

Data are expressed as mean ± standard deviation and were statistically analysed using Prism software (GraphPad Software, San Diego, CA, USA). Data were subjected to unpaired two-tailed *t*-tests or two ANOVA with Tukey’s multiple comparison test, and significance was set at *p* < 0.05 [27].

## 3. Results

### 3.1. ZNS Inhibited mitROS Generation in the Microglia of In Vivo PD Models

Because aggravating immune responses play a central role in the pathogenesis of PD, the appropriate control of the immune system may be more important in the therapeutic view of this disease. Based on this concept, we evaluated whether ZNS induces any effects on microglial cells of a brain with PD. Considering the inflammatory features of a brain with PD, we developed a special inflammatory mouse PD model by challenging with two neurotoxins, MPTP and LPS, in which mice received MPTP for 5 days. Thereafter, these mice continued to receive LPS for 3 days (Figure 1A). Using flow cytometry, we gated microglial cells for microglial mitROS generation analysis, where ZNS exhibited suppressive effects. However, ZNS post-treatment did not significantly affect the number and morphological features, as shown by the dot plot (Figure 1B,C).

### 3.2. ZNS Abolished mitROS Generation and Phagocytic Activity in LPS-Treated BV2 Cells

**Figure 2:**
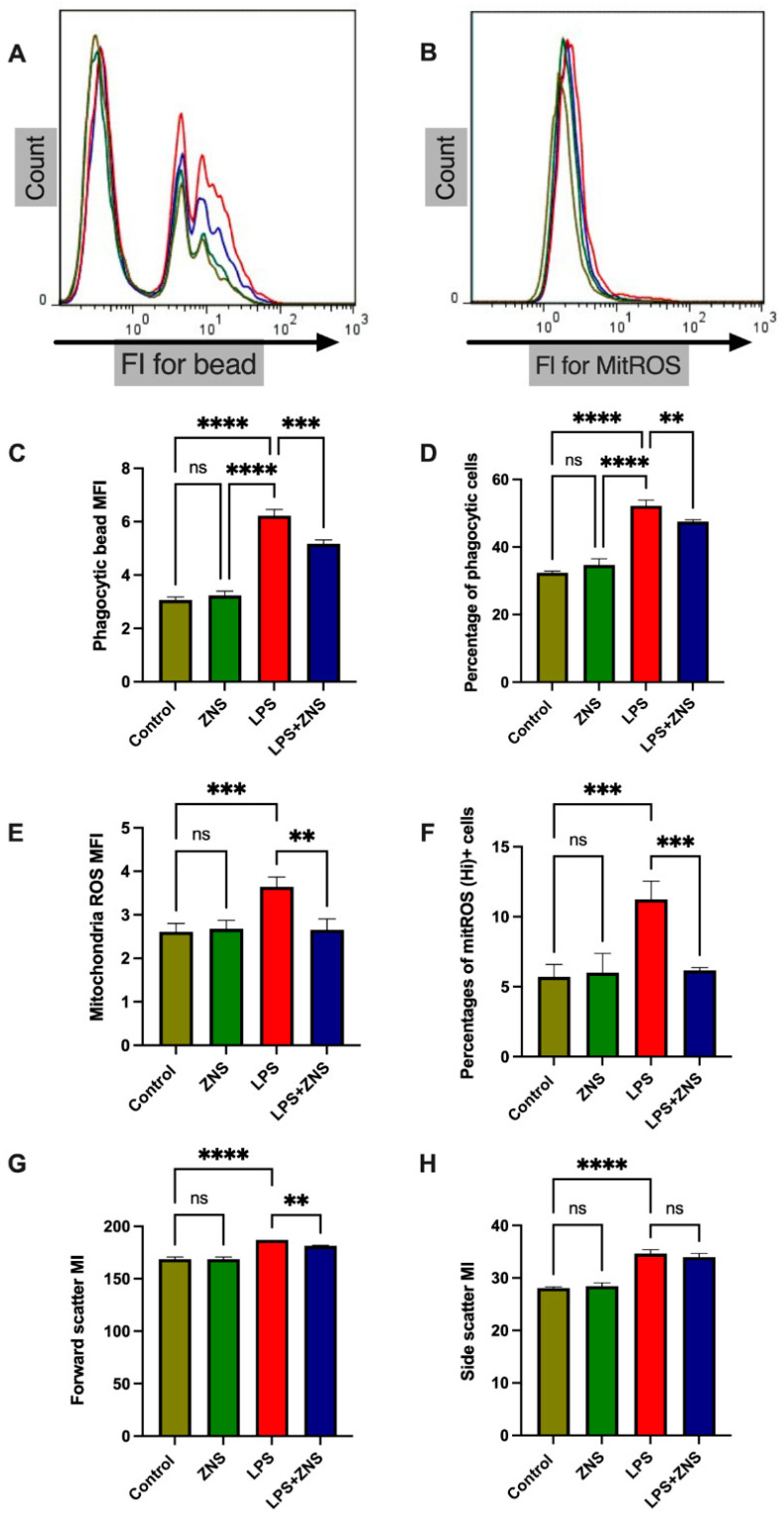
ZNS inhibits the phagocytic activity and mitochondrial reactive oxygen species generation of LPS-treated BV2 (murine microglial cell line). Cells were treated with or without LPS (1 µg/mL) and co-treated with or without ZNS (100 µM) overnight. (**A**) Representative histogram for phagocytic beads, (**B**) representative histogram for mitROS, (**C**) mean fluorescence intensity (MFI) for phagocytic bead, (**D**) percentages of phagocytic cells, (**E**) MFI for mitROS, (**F**) percentages of mitROS (high) positive cells, (**G**) mean intensity (MI) for forward scatter values, and (**H**) MI for side scatter values. Histogram peak colours: asparagus green for control, fern green for ZNS, maraschino red for LPS, and midnight blue for LPS + ZNS. Data are expressed as mean ± SD; (n-3). Asterisks indicate ** 0.01, *** 0.001, **** 0.0001 by two-way ANOVA. LPS, lipopolysaccharide; mitROS, mitochondrial reactive oxygen species; ZNS, zonisamide; SD, standard deviation.

Next, we assessed the effects of ZNS in BV2 mouse microglial cells in vitro. LPS exposure increased mitROS production and forward and side scatter values; however, when LPS was co-exposed with ZNS, the effects of LPS on mitROS were partially abolished (Figure 2B,E,F). Indeed, ZNS did not inhibit the effects of LPS on the side scatter value of BV2 cells, but it partially inhibited the effects of LPS on the forward scatter value of the cells (Figure 2G,H).

Excessive microglial phagocytosis in dopaminergic neuronal cells in the substantia nigra is considered one of the most important pathological events in PD progression. In the aspect of microglia phagocytosis, CD68 expression in brain tissues is an essential indicator of microglial phagocytosis [6]. In one of our previous studies, we showed that bromovalerylurea exhibited antiparkinsonian effects by inhibiting CD68 expression [23]. Similarly, another recent study demonstrated that a combined treatment of 1-deoxynojirimycin and ibuprofen decreased microglial phagocytosis and protected dopaminergic neuronal degeneration [28]. In this study, we demonstrate another antiparkinsonian feature of ZNS, which lowered the phagocytic activity of LPS-treated BV2 murine microglial cell lines (Figure 2A,C,D).

### 3.3. ZNS Ameliorated Mitochondrial Dysfunction of LPS-Treated BV2 Cells but Not MPP^+^-Treated Neuro 2A Cells

**Figure 3:**
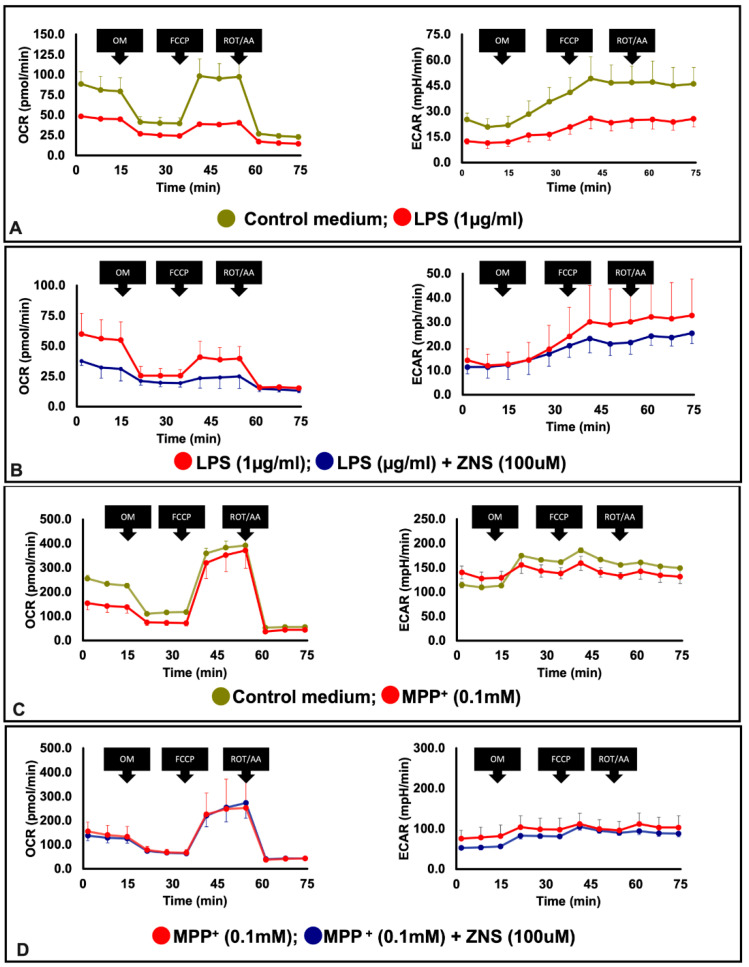
Ameliorative effect of ZNS on mitochondrial dysfunctions in LPS-induced in BV2 cells. (**A**) Cells were seeded in a Seahorse XF plate at 2.5 × 10^4^ cells per well and treated with or without LPS (1 µg/mL) overnight. (**B**) Cells were treated with LPS in combination with or without ZNS overnight. (**C**) Cells were plated in a Seahorse XF plate at 2.5 × 10^4^ cells per well and treated with or without MPP^+^ 100 µM for 1 h. (**D**) Cells were treated with MPP^+^ in combination with or without ZNS. The results are representative data from least three independent experiments. Representative bioenergetics profile data (n = 3) are shown as means ± SD, oxygen consumption rate (OCR, left) and extracellular acidification rate (ECAR, right). LPS, lipopolysaccharide; OM, oligomycin; FCCP, carbonyl cyanide-p-trifluoromethoxyphenylhydrazone; ROT/AA, rotenone and antimycin; ZNS, zonisamide; SD, standard deviation.

Accumulating evidence has shown that impaired mitochondrial biogenesis is strongly linked to the pathogenesis of PD [29]. Using BV2 microglia cell lines, we prepared an in vitro PD model in which these cells were LPS-challenged. Of particular interest in this inflammatory PD model, LPS exposure decreased mitochondrial OCR and ECAR (Figure 3A). ZNS significantly prevented the development of the depressive effects of LPS on OCR (Figure 3B). Based on the effect of ZNS on BV2 cells, we extended our study to neuronal cells. In this approach, we used a murine neuronal cell line Neuro 2A, and used MPP^+^ to prepare an additional in vitro cellular model of PD. Similar to LPS, MMP^+^ exposure decreased mitochondrial OCR and ECAR in Neuro 2A cells (Figure 3C). However, in this case, ZNS did not exhibit any preventive effects (Figure 3D).

### 3.4. ZNS Reversed LPS Gene Expression in Treated BV2 Cells

**Figure 4:**
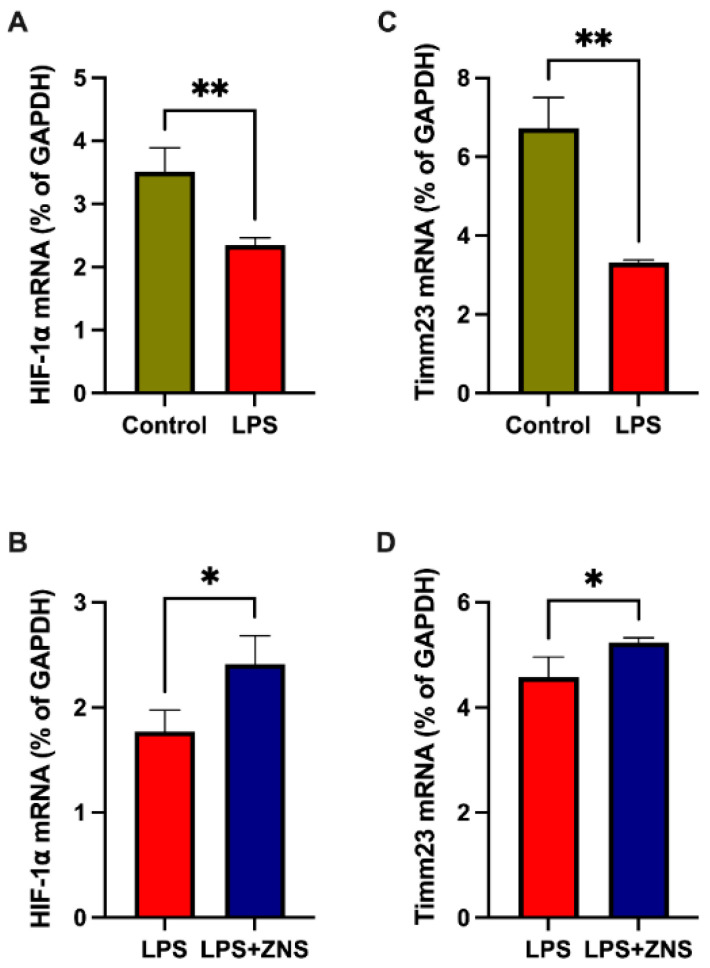
Ameliorative effect of ZNS on LPS-induced mitochondrial dysfunction-related genes in BV2 cells. (**A**) and (**C**) Cells were treated with or without LPS (1 µg/mL) overnight. (**B**) and (**D**) Cells were treated with LPS in combination with or without ZNS. mRNA expression of hypoxia inducible factor 1 α (HIF1α) (**A**) and (**B**), Timm23 (**C**) and (**D**). Data are expressed as mean ± SD. LPS, lipopolysaccharide; ZNS, zonisamide; SD, standard deviation. Asterisks indicate * 0.05, ** 0.01, by student *t*-test.

Several studies have shown that the stabilisation of the hypoxia-inducible factor 1 α (HIF1α) plays a role in neuroprotection in PD brains [30]. Our qPCR data showed that treatment with LPS decreased the expression of mRNA encoding HIF1α, and ZNS partially abolished this LPS effect on BV2 cells (Figure 4A,C). Next, we assessed Timm23 mRNA expression, which has been shown to attenuate MPTP-induced denervation at the level of dopaminergic cell bodies in the substantia nigra pars compacta [31]. As shown in an endothelial cell study published earlier [32], we found that LPS treatment downregulated the expression of mRNA encoding Timm23 (Figure 4B). Similar to HIF1α, ZNS ameliorated the expression of Timm23 mRNA (Figure 4D). These data suggest that ZNS has ameliorative effects on microglial dysfunction in PD.

## 4. Discussion

Currently in Japan, ZNS is considered to be an adjunctive antiparkinsonian drug because of its beneficial effects on motor and sleep problems in patients with PD [13]. The antiparkinsonian effects of ZNS have been reported in our previous studies on MPTP and in a 6-OHDA-treated animal model of PD, where we found that ZNS acts as a neuroprotectant against MPTP-induced dopaminergic neuronal degeneration by acting directly on neurones and astrocytes [14,15,16,17]. In addition to our studies, several research studies published by different groups have highlighted the antiparkinsonian effects of ZNS [33]. A study showed that ZNS reduced neuroinflammation by inhibiting Nav1.6 and TNFα in microglial cells in an MPTP-treated mouse model of PD. The expression of Nav1.6 in microglial cells was found to be increased in patients with PD [18]. Microglial cells are believed to be involved in the progressive loss of dopaminergic neurones in PD through the release of potentially harmful substances. The depletion of the microglia with GW2580 (a CSF-1R inhibitor) attenuated MPTP-induced dopaminergic neuronal loss and motor behavioural deficits in a PD model [8]. We sought to determine whether ZNS has any effect on the remodelling of microglial cells in the LPS-primed MPTP murine model of PD. Our data revealed that ZNS inhibits mitROS generation by microglia in in vivo and in vitro PD models. MitROS is involved in microglial inflammatory responses by activating mitogen-activating proteins (MAPs), as pharmacological inhibition of mitROS suppresses the activation of MAPs, NF-κB nuclear translocation, and TNFα release [34]. Related cellular and animal studies have demonstrated that NADPH oxidase is the main contributor to microglial ROS [35,36]. The glycoprotein gp91^phox^ is a critical catalytic subunit of NADPH oxidase, which modulates dopaminergic neuronal degeneration by releasing ROS and cytokines in the brain [37,38]. Increased NADPH oxidase in microglial cells has been documented in post-mortem studies, where an increased expression of gp91^phox^ has been observed [39]. Moreover, considering the inhibitory effects of ZNS on the expression of F4/80, a mature phagocytic cell marker in MPTP-treated mice [18], we identified the inhibitory effects of ZNS on the phagocytic activity of microglial cells.

In PD pathogenesis, mitochondrial dysfunction is characterised by overwhelmed oxidative stress, lack of respiratory chains, and defective mitophagy flux [40]. During inflammation, the maintenance of the normal mitochondrial function is critical for skewing from M1 type macrophages to M2 macrophages [41]. LPS is considered a potent M1 inducer, and exposure of LPS to BV2 suppressed mitochondrial bioenergetics, as revealed by the decreased OCR and ECAR. This is consistent with the findings of another study in which exposure of murine macrophages to LPS reduced both OCR and ECAR [42]. In terms of mitochondriopathy, as previously pointed out in striatal neurones, ZNS showed neuroprotective effects against mitochondrial impairment through complex I conservation [43]. In the present study, co-exposure to ZNS with LPS partially abolished the inhibitory effect of LPS on microglial OCR. However, similar to LPS-treated BV2 cells, MPP^+^-treated Neuro 2A cells showed reduced mitochondrial OCR and EACR, but ZNS was not deemed to be effective in terms of reversing the respective MPP^+^ effects in our present studies. A possible explanation for these apparently contradictory results could be attributed to variations in cellular models.

Patients with PD showed decreased expression of HIF1α, which is part of a highly conserved complex that governs the expression of several neuroprotective factors involved in cellular stress responses [30]. In the present study, LPS reduced the mRNA expression of HIF1α, whereas ZNS reversed this effect. The expression of another protein involved in the protein import machinery, Timm23, was found to be decreased in patients with PD, and mitochondrial complex I inhibition with MPP^+^ also reduced the expression of Timm23 [31]. ZNS was found to be effective for mitochondrial complex I [43], and in the present study, ZNS partially reversed the mRNA expression of Timm23 in LPS-treated microglial cells.

Altogether, these findings underline the efficacy of ZNS as an antiparkinsonian drug because it was found to protect neurones in inflammatory PD brains by inhibiting mitROS generation and remodelling defective or detrimental microglia to supportive or beneficial ones. Therefore, these results suggest that ZNS may induce profound mitochondrial effects on related microglial dysfunction, and thus modify the risk of rapid PD progression.
